# The Psychosocial Consequences of Prostate Cancer Treatments on Body Image, Sexuality, and Relationships

**DOI:** 10.3389/fpsyg.2021.765315

**Published:** 2021-10-22

**Authors:** Joanna M. Mainwaring, Lauren M. Walker, John W. Robinson, Richard J. Wassersug, Erik Wibowo

**Affiliations:** ^1^Department of Anatomy, School of Biomedical Sciences, University of Otago, Dunedin, New Zealand; ^2^Division of Psychosocial Oncology, Department of Oncology, Cumming School of Medicine, University of Calgary, Calgary, AB, Canada; ^3^Department of Cellular & Physiological Sciences, University of British Columbia, Vancouver, BC, Canada

**Keywords:** prostate cancer, sexual side effects, body image, physical activity, gay and bisexual men (GBM), psychosocial counseling

## Introduction

Body image can have an important impact on quality of life, particularly in the context of cancer. Body image is considered the “*attitudes and perceptions about the body, particularly its appearance*” (Tylka, 2018) including “*thoughts, beliefs, feelings, and behaviors*” pertaining to one's body (Cash, 2004). Body image is often impacted by cancer and its treatment.

In the context of prostate cancer (PCa), how men view their body can change as a result of treatment side effects. The impact of PCa treatments on men vary with cancer stage and treatment. Localized PCa is regularly treated with radical prostatectomy (surgical removal of the prostate, seminal vesicles, and proximal lymph nodes) or radiation therapy. Such treatments are known to have side effects that significantly impact sexual function, urinary and even bowel control (Sun et al., 2014; Downing et al., 2019). In the case of cancer recurrence, patients with systemic or locally advanced PCa are regularly treated with androgen deprivation therapy (ADT). ADT is also used as neo-adjuvant therapy along with radiotherapy.

ADT controls PCa growth by greatly suppressing testosterone in the body and therefore has profound physical, sexual, and psychological side effects (Smith, 2007; Downing et al., 2019; Wibowo et al., 2019). Considering the low mortality rate of PCa (Lu-Yao et al., 2008; Hamdy et al., 2016), many patients endure treatment side effects for years and sometimes decades. Some of these side effects, including physical and sexual effects, can directly affect body image and can have negative psychological and social implications.

## Effects of Primary Prostate Cancer Treatments on Body Image

How PCa treatments affect the body and bodily functions depends on the focal nature of the treatment and impact on neighboring structures. For example, due to the proximity of the bladder to the prostate, incontinence and urine leakage are common side effects of a prostatectomy and, to a lesser extent, radiation therapy (Sun et al., 2014). Incontinence can be humiliating and can decrease men's quality of life (Stewart et al., 2003; Powel and Clark, 2005; Downing et al., 2019); as can management strategies that include the use of pads and other absorbent undergarments (Palmer et al., 2003). These lifestyle changes may in turn lower patients' self-confidence and make them withdraw socially (Bowie et al., 2021).

Climacturia (urination during orgasm) is a side effect mainly associated with prostatectomy (Lee et al., 2006). Climacturia has substantial negative effects on men's sexual lives and overall quality of life (Nilsson et al., 2011). Urinary leakage during sexual activity can be bothersome to both patient and partner, resulting in embarrassment, and act as a significant deterrent to willingness to engage in sexual activity (Mendez et al., 2018).

Erectile dysfunction due to PCa treatment has a profound impact on sexual satisfaction and reduces sexual confidence and positive feelings about one's body. Other PCa treatment side effects that are common but often neglected by healthcare providers include reduced ejaculation (Akbal et al., 2008), anorgasmia (Hollenbeck et al., 2003), and decreased orgasm intensity (Koeman et al., 1996; Frey et al., 2017). Various management strategies are available for erectile dysfunction (Sherer and Levine, 2014; Elliott and Matthew, 2017; Salonia et al., 2017; Wassersug and Wibowo, 2017). Nevertheless, many men report feeling less manly due to erectile dysfunction following PCa treatment (Tsang et al., 2018b), and need to find new ways to be sexually intimate that are not erection-dependent (Gray et al., 2002; Wassersug et al., 2016; Duthie et al., 2020).

A significant weakness of the literature on PCa and masculinity is that many authors have reported that PCa treatments result in a loss of “masculinity” without rigorously defining what masculinity is. Still, most recent reports suggest that patients perceive that loss of “masculinity” as a result of impairment in bodily functions; i.e., in erectile function and overall physical strength (Tsang et al., 2018a,b).

## Effects of Androgen Deprivation Therapy on Body Image

Patients who are treated with ADT are known to have poorer body image than ADT-naïve patients (Harrington et al., 2009), and this may be for a variety of reasons. Perhaps most significantly, side effects associated with the loss of testosterone include a vast array of physical changes such as fatigue, weight gain, muscle loss, breast enlargement, genital shrinkage, and body hair loss (Nguyen et al., 2015; Wibowo et al., 2019). Many of these effects can be perceived as emasculating for men (Gentili et al., 2019), with erectile function being definitional to most PCa patients' understanding of what it means to be “a man” (Muermann and Wassersug, 2021). While the magnitude of many of the adverse side effects of PCa treatment that impact body image can be reduced with physical exercise, the challenge remains that poor body image following treatment can conversely be a barrier for exercise, especially exercise with others (Gentili et al., 2019).

Sexual implications of ADT can also impact body image. ADT commonly leads to a loss of sexual desire (Fode and Sonksen, 2014). If patients had not received a primary treatment for localized disease before ADT, they would now experience substantial and abrupt sexual changes including erectile dysfunction, reduced orgasmic capability if not total anorgasmia, diminished ejaculation, and reduced sexual interest, which can lower their body image (Tsang et al., 2018b). Data from healthy young men demonstrate a relationship between negative genital self-image and sexual dissatisfaction (Van Den Brink et al., 2018). The genital shrinkage associated with prolonged ADT can be very distressing to patients and further impact genital and overall self-image. While patients on ADT have less frequent sexual activity (Walker et al., 2018), the precise relationship between body image and sexual activity in men on ADT remains to be determined.

ADT may also lead to psychological changes in PCa patients' mood. ADT is associated with an increased risk of depression (Nead et al., 2017), anxiety (Dinh et al., 2017), and emotional lability (Donovan et al., 2015) as exemplified typically by increased spontaneous tearfulness (Galvin et al., 2019). Currently, it is unclear how psychological changes following ADT influences patient's body image. In the general population, depression and higher body weight are associated with poor body image (Silva et al., 2019). ADT patients are at elevated risk for sarcopenic obesity, and their increase in weight may potentially be a contributing factor to their depression and poor body image.

Not unexpectedly, the psychological implications of altered body form and function following ADT can vary with gender identity. Previously, Wassersug and Gray (2011) reported that some male-to-female transgender do not perceive changes induced by testosterone suppression for gender affirmation as negatives. For example, some of the distressing changes that cis-gendered males experience as emasculating—notably loss of muscle mass, gynecomastia, loss of body hair—are experienced as gender affirming and thus may enhance the body image of some transgender women.

## Body Image Concerns of Gay and Bisexual Men Treated for Prostate Cancer

The burden of PCa treatments on gay and bisexual men (GBM) has received substantial research in recent years (Dowsett et al., 2014; Ussher et al., 2016a, 2019; Wassersug et al., 2016), however body image has not yet been rigorously studied in the GBM PCa population. GBM without PCa are likely to have poorer body image (Gil, 2007) and more body dissatisfaction (Kaminski et al., 2005; Tiggemann et al., 2007) than straight men. As such, PCa side effects may differentially affect GBM. GBM with PCa are also less likely than straight patients to be in a committed relationship (Wassersug et al., 2016) and more likely to have casual sexual relationships (Ussher et al., 2019). Thus, the impact of changes in body image induced by PCa treatment may be particularly burdensome for GBM when it comes to dating and seeking new sexual relationships. Sexual dysfunction has been identified as a major deterrent for attempting to establish new intimate relationships for single PCa patients in general (Mathew et al., 2020). It remains to be determined if this problem is more severe for non-heterosexual men.

To date, only one study (Thomas et al., 2018) has explored body image in GBM and straight men using a validated scale, namely the Multidimensional Body-Self Relations Questionnaire (Cash, 2000). On three subscales of this survey instrument, no differences were observed in appearance evaluation, health evaluation, or health orientation. However, the authors excluded several subscales from this questionnaire (e.g., overweight preoccupation, body area satisfaction, fitness evaluation, appearance orientation) which may vary between GBM and straight PCa patients, especially given the profound bodily changes associated with ADT (Elliott et al., 2010). Given the research on GBM without PCa, it may be reasonable to assume that many components of body image are more affected in GBM than in straight men.

Overall, sexual changes due to PCa treatment are comparable between GBM and straight men (Ussher et al., 2016a; Thomas et al., 2018). However, GBM tend to be more distressed by sexual dysfunction than straight men (Ussher et al., 2016a). This may in part be due to the inability to engage in anal sex among GBM when erectile dysfunction is present. Firmer erections are needed for anal than vaginal penetration (Goldstone, 2005; Ussher et al., 2016b), thus even minor reductions in erectile rigidity may make anal penetration impossible for GBM burdened by PCa sexual side effects. GBM with PCa conversely may also be concerned about rectal bleeding and rectal pain following radiation treatment if they take on the receptive role (Blank, 2005; Ussher et al., 2016b). Furthermore, the loss of ejaculation has been noted to be more bothersome to GBM than straight men (Wassersug et al., 2013). In sum, reduced sexual capacity, loss of sexual function and bodily discomfort amongst PCa survivors, may differentially affect GBM even more than straight men.

## Assessment and Improvement of Body Image

More studies directly assessing body image as a treatment outcome in PCa patients are warranted. A standard, concise, well-validated, and commonly used assessment instrument is the Body Image Scale, developed by Hopwood et al. (2001) and modified by Mcdermott et al. (2014). This is a 9-item self-report questionnaire that could easily be added to questionnaire batteries assessing psychological or relational outcomes among PCa patients.

Although we have briefly reviewed the potential impacts of sexual and physical affects from PCa treatments on body image, the relationships between body image and sexuality—specifically for men treated for PCa—is a topic worth more in-depth study. Relevant to the PCa population, which is largely made up of older men, is the fact that aging itself can erode body image and that in turn can undermine the experience of sexual pleasure (Schwartz et al., 2014). Body image is known to have a significant negative relationship with sexual satisfaction and sexual function for women (Afshari et al., 2016; Quinn-Nilas et al., 2016), but this has been under-investigated for men and, to our knowledge, not studied within men treated for PCa.

Interventions to improve body image are available, but require evaluation in the context of PCa. For example, moderate-to-vigorous aerobic exercise has been reported to improve PCa patients' sense of their manhood [assessed *via* the Personal Attributes Questionnaire (Helmreich et al., 1981)] and body image [assessed *via* the Body Image Scale (Langelier et al., 2018)]. However, the association between exercise and body image was found only in patients not undergoing ADT. It remains unclear why this was not the case for men on ADT. Psychosocial counseling may also be a promising strategy to improve body image. Though we are not aware of any studies specific to PCa, psychosocial counseling and psycho-education have been shown to improve body image in women with breast cancer (Lewis-Smith et al., 2018).

## Conclusion

In conclusion, the physical and sexual side effects of PCa treatments may lower men's body image. This change may affect patients' sexual intimacy with their partners, as well as the confidence of unpartnered patients in forming a new relationship.

It remains to be determined whether PCa treatment causes more profound changes in the body image of GBM than straight men. While research in this area is limited, exercise programs and psychosocial counseling may potentially benefit PCa patients who have concerns about their body image.

## Author Contributions

JM and EW: writing first draft of the manuscript. JM, LW, JR, RW, and EW: manuscript editing and concept and discussion. All authors contributed to the article and approved the submitted version.

## Funding

Salary supported for LW was provided by the Daniel Family Chair in Psychosocial Oncology. Our research within the Androgen Deprivation Therapy Educational Program (www.LIFEonADT.com) was supported by an unrestricted educational grant from Astellas to the Prostate Cancer Center in Calgary, AB, Canada.

## Conflict of Interest

The authors declare that the research was conducted in the absence of any commercial or financial relationships that could be construed as a potential conflict of interest.

## Publisher's Note

All claims expressed in this article are solely those of the authors and do not necessarily represent those of their affiliated organizations, or those of the publisher, the editors and the reviewers. Any product that may be evaluated in this article, or claim that may be made by its manufacturer, is not guaranteed or endorsed by the publisher.
